# Nanopore metatranscriptomics reveals cryptic catfish species as potential *Shigella flexneri* vectors in Kenya

**DOI:** 10.1038/s41598-022-17036-y

**Published:** 2022-08-16

**Authors:** Andrew J. Tighe, Sean Grayson, John Byrne, Sanni Hintikka, Lisa Jessen, Jake Dempsey, Lauren Browne, Mary Kelly-Quinn, Bernerd Fulanda, Neil M. Ruane, Jens Carlsson

**Affiliations:** 1 Area 52 Research Group, School of Biology and Environmental Science/Earth Institute/University College Dublin, Dublin 4, Ireland; 2 Fish Health Unit, Marine Institute, Oranmore, Co., Galway, Ireland; 3 School of Biology and Environmental Science/Earth Institute, University College Dublin, Dublin 4, Ireland; 4 Department of Biological Sciences, Pwani University, Kilifi, Kenya; 5 Fisheries Ecosystems Advisory Services, Marine Institute, Oranmore, Co., Galway, H91 R673 Ireland

**Keywords:** Pathogens, Food microbiology, RNA sequencing

## Abstract

Bacteria in the *Shigella* genus remain a major cause of dysentery in sub-Saharan Africa, and annually cause an estimated 600,000 deaths worldwide. Being spread by contaminated food and water, this study highlights how wild caught food, in the form of freshwater catfish, can act as vectors for *Shigella flexneri* in Southern Kenya. A metatranscriptomic approach was used to identify the presence of *Shigella flexneri* in the catfish which had been caught for consumption from the Galana river. The use of nanopore sequencing was shown to be a simple and effective method to highlight the presence of *Shigella flexneri* and could represent a potential new tool in the detection and prevention of this deadly pathogen. Rather than the presence/absence results of more traditional testing methods, the use of metatranscriptomics highlighted how primarily one SOS response gene was being transcribed, suggesting the bacteria may be dormant in the catfish. Additionally, *COI* sequencing of the vector catfish revealed they likely represent a cryptic species. Morphological assignment suggested the fish were widehead catfish *Clarotes laticeps*, which range across Africa, but the *COI* sequences from the Kenyan fish are distinctly different from *C. laticeps* sequenced in West Africa.

## Introduction

Zoonotic pathogens pose an ongoing threat to human society, as acutely highlighted with SARs-COV2, the causative agent of Covid-19^1^. Generally, zoonotic viruses originate in mammalian or avian hosts, with no known zoonotic viruses originating in fish^2^. However, fish can be sources of a number of bacterial and parasitic diseases^3,4^. The primary pathogens from fish that pose a risk to humans are bacterial, with pathogens such as *Salmonella* sp., *Campylobacter* sp., *Escherichia coli*, *Listeria monocytogenes* and *Yersinia* sp. being responsible for most foodborne outbreaks from fish worldwide^5^. It is likely also that undescribed zoonotic pathogens are circulating in wild fish, with developing countries in the tropics predicted to be the highest risk areas for future zoonoses to emerge^6^. Therefore in this paper, a pilot study for an early warning system using shotgun sequencing approach was used to determine whether any pathogens could be identified from wild caught widehead catfish *Clarotes laticeps* found in the Galana river in South East Kenya. *Clarotes laticeps* are an important protein source for local communities and are routinely harvested directly from the river. The species is a tropical, freshwater catfish in the family *Claroteidae*^7^, which is present in all Nilo-Sudanese basins^8^ in addition to East Africa and the Nile^9^.

A number of bacterial pathogens are known to be associated with the consumption of catfish species (both farmed and wild caught), such as *Salmonella*, *Campylobacter*, *E. coli*, *L. monocytogenes*, *Staphylococcus aureus* and *Vibrio*^3^. Some bacteria, such as *Salmonella*^10–12^ and *Campylobacter*^13^ are thought to be introduced to aquaculture ponds through the faeces of birds and wildlife, and faecal matter from ruminants is known to contaminate ponds with pathogenic *E. coli*^3^. Additionally, *Salmonella* has been detected in wild catfish^14^ and *Grimontia hollisae* (formerly known as *Vibrio hollisae*) was implicated as the cause of septicaemia in a man who had eaten wild caught catfish in the USA^15^. In Sub-Saharan Africa, wild caught fish have been shown to be sources of bacterial enteropathogens, with one example in Central African Republic specifically linking the presence of Shiga toxin producing *E. coli* in wild fish and river water to the upstream slaughter of zebu cattle *Bos indicus*^16^.

Enterotoxigenic *E. coli* in addition to *Shigella* spp. are responsible for the majority of bacterial caused diarrhoeal episodes in children under 5 years in Sub-Saharan Africa^17^. Similar to *E. coli*, *Shigella* spp. are also known to be introduced to water sources from anthropogenic sources such as untreated sewage and agricultural runoff^18^. Outbreaks of shigellosis due to *Shigella* spp. from contaminated water are known throughout Sub-Saharan Africa^19^, in addition to Asia^18,20^. Additionally the presence of antimicrobial resistance genes has been detected in *Shigella* spp. in waterways, likely due to pollution in water sources from industry, veterinary medications and human medical treatment^18^.

The detection of bacterial pathogens, both from fish and other sources, has increasingly been based on the use of polymerase chain reaction (PCR) based approaches over the past two decades^21^. One significant drawback of this approach is that it can only be used to test for known pathogens, with multiple presence/absence tests needed to check for all potential pathogens. Second and third generation sequencing platforms such as Illumina’s MiSeq and Oxford nanopore’s MinION, however, allow for shotgun sequencing approaches which can be used to sequence all DNA present in a sample, potentially revealing most microbial life present in one test, saving time and allowing for the rapid discovery of novel disease causing pathogens. Third generation nanopore sequencing has been used in a number of shotgun metagenomic studies to identify pathogens, such as diagnosing the cause of infections in orthopaedic implants^22^, identifying oral bacteriophages^23^ and characterising the microbiomes of preterm human babies^24^. The approach has also been used to identify waterborne pathogens, with Reddington et al.^25^ showing how wastewater influents increased the abundance of *Arcobacter* when compared to cleaner parts of the Havelse river in Denmark.

In this study, a shotgun transcriptomics approach was used in an attempt to identify pathogens found in wild widehead catfish in the Galana river in South-East Kenya. In addition to shotgun sequencing, the *cytochrome oxidase I* (COI) gene of the fish was sequenced using Sanger sequencing, in order to confirm the species of catfish found in the river. Although the widehead catfish in the Galana river is currently classified as *C. laticeps*, there remains taxonomic uncertainty as to whether this population of catfish are in fact *C. laticeps*^26^.

## Methods

### Sample collection and sequencing

**Figure 1:**
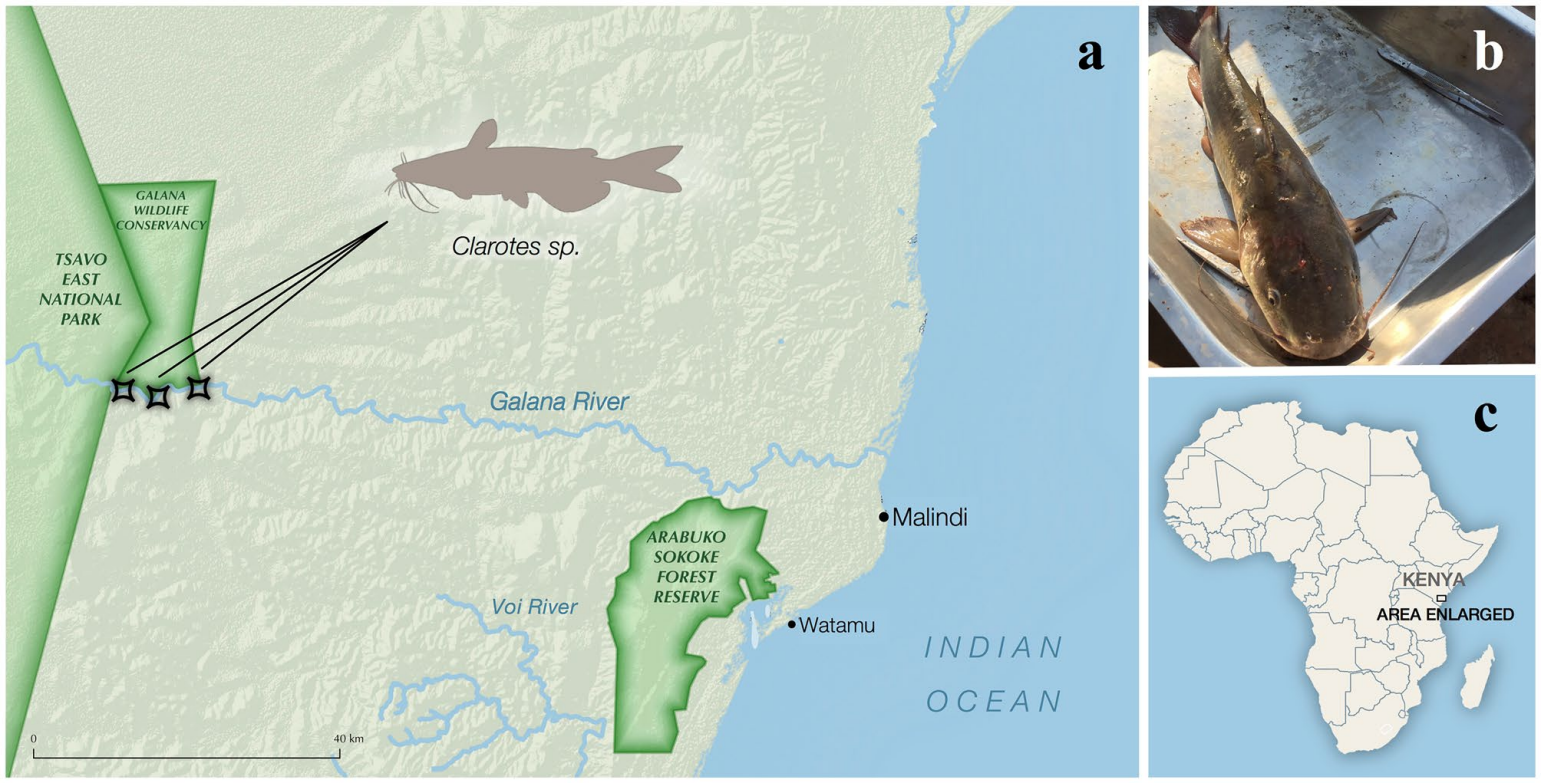
Locations where samples were collected. (**A**) Map showing were the fish were collected in South East Kenya. (**B**) Photograph of one of the widehead catfish provided by the scouts. (**C**) Map highlighting the location of the region shown in (**A**) on the African continent. Map generated using QGIS3.6 (https://qgis.org/en/site/forusers/download.html).

Four catfish samples were collected between the 7th and 13th of August, 2019 from three points along the Galana river (Fig. 1). The deceased catfish, which had been caught using a baited hook and line, were gifted by local scouts and samples were taken from remains prior to preparation as food. The catfish sampled were identified as widehead catfish based on morphological characteristics. Fish were dissected in a sterile metal tray, with utensils sterilised by ethanol and flamed between samples. Single muscle and heart samples were taken from each fish, but in the process of dissecting the fish the intestines were significantly nicked, meaning the heart samples were likely contaminated with intestinal material. Tissue samples were placed in RNA*later* (Invitrogen, USA) in 2 mL O-ring tubes and kept at ambient temperature in the field for up to eleven days, and later stored at − 80 °C.

The four heart samples were homogenised using a TissueLyser (Qiagen), and RNA was then extracted using an QIAamp Viral RNA Mini Kit (Qiagen) via a QiaCube extraction machine (Qiagen). The RNA was converted to cDNA using Superscript II Reverse Transcriptase (Invitrogen, USA) following the manufacturer’s guidelines using 8 μl of RNA and 1 μl random hexamer primers per reaction.

The concentration of the RNA/DNA samples for four samples was determined using a BioDrop μLite v1.0.4 (Thermo Fisher Scientific) and 7.5 μl of each sample was barcoded using 2.5 μl of Fragmentation Mix RB01-04 from Oxford Nanopore’s Rapid Barcoding Sequencing kit (SQK-RBK004, Oxford Nanopore Technologies). Barcoded samples were then equimolar pooled and sequencing adapters were ligated to the DNA library using 4 μl of Rapid Adapter reagent (RAP, Oxford Nanopore Technologies). The barcoded library was loaded onto a MinION flow cell Mk1 R9.4.1 (Oxford Nanopore Technologies) and run via MinKNOW software v.3.6.5 (Oxford Nanopore Technologies) without real-time base-calling for 68 h on a MinION sequencer (Oxford Nanopore Technologies).

### Nanopore data analysis

Guppy v.3.2.10 (Oxford Nanopore Technologies) was used to base-call the output from the MinION sequencing run. Porechop (https://github.com/rrwick/Porechop) was used to remove the adaptor sequences from the MinION sequence data. Porechop was also originally used to sort samples by barcodes, but as 215,028 reads were left unassigned (89.44% of reads) and due to the low read depth it was decided to pool the reads for all four catfish samples for further analysis.

In order to first assess the microbial organisms present, the fastq reads were processed using the What’s In My Pot (WIMP) workflow on Oxford Nanopore’s EPI2ME platform (https://epi2me.nanoporetech.com/, Oxford Nanopore Technologies). Following the WIMP analysis, a reference fasta database was generated using an existing genome for North American yellow catfish *Tachysurus fulvidraco* (Genbank Assembly Accession: GCA_003724035.1) to act as a proxy for the host genome. Additionally a genome for the parasite *Schistosoma haematobium* (Genbank Assembly Accession: GCA_000699445.2), the 12,642 existing viral sequences from ray-finned fish *Actinopterygii* hosts available on the NCBI virus database (https://www.ncbi.nlm.nih.gov/labs/virus/vssi/#/) and the genomes for the 23 bacterial and fungal species identified through WIMP (Table 1) were added to the reference fasta file. The fastq reads were mapped against the reference fasta database using the NanoPipe web server (http://www.bioinformatics.uni-muenster.de/tools/nanopipe2/index.hbi?)^27^. The consensus sequences for mapped reads were then subject to a BLASTn search (https://blast.ncbi.nlm.nih.gov/Blast.cgi), and consensus sequences which did not correspond to any existing sequences were removed from the final result.

**Table 1:** Microbial genomes included in the reference database.

Species	Genbank Assembly Accession
*Candida dubliniensis*	GCF_000026945.1
*Acinetobacter baumannii*	GCF_002116935.1
*Aquicella siphonis*	GCF_902459484.1
*Arcobacter cibarius*	GCA_013372265.1
*Chryseobacterium lactis*	GCA_003815875.1
*Clostridium perfringens*	GCA_000013285.1
*Cutibacterium acnes*	GCA_000008345.1
*Escherichia coli*	GCA_904425475.1
*Enterococcus faecium*	GCA_010120755.1
*Flavobacterium gilvum*	GCA_001761465.1
*Klebsiella pneumoniae*	GCA_000240185.2
*Metabacillus litoralis*	GCA_003667825.1
*Moraxella osloensis*	GCA_001553955.1
*Nodularia spumigena*	GCA_000340565.3
*Pseudomonas synxantha*	GCA_003851495.1
*Salmonella enterica*	GCA_001558355.2
*Sphingobacterium daejeonense*	GCA_901472535.1
*Sphingomonas paucimobilis*	GCA_003314795.2
*Staphylococcus aureus*	GCA_000013425.1
*Vibrio parahaemolyticus*	GCA_004006515.1
*Shigella flexneri*	GCA_013391345.1
*Escherichia coli*	GCA_000183345.1
*Sugiyamaella lignohabitans*	GCA_001640025.2

List of species genomes downloaded from Genbank based on the results generated from WIMP

### Sequencing and analysing the *COI* of the host

A separate DNA extraction was performed on the catfish muscle samples collected, with samples being incubated at 56 °C for 2 h in a mix of 12 µl of proteinase K (20 mg/ml) and 400 µl of 10% Chelex solution, followed by 15 min at 99 °C. Samples were then centrifuged for 1 min at max speed (20,817 G) and 150 µl of DNA supernatant was placed in a new 1.5 mL Eppendorf tube. A 653 bp amplicon of the *COI* gene was amplified using the primer pair FishF2 (5’- TGT AAA ACG ACG GCC AGT CGA CTA ATC ATA AAG ATA TCG GCA C -3’)^28^ and FishR2 (5’- CAG GAA ACA GCT ATG ACA CTT CAG GGT GAC CGA AGA ATC AGA A -3’)^28^. A 25 µl master mix was prepared in a UV-sterilized hood consisting of 3.125 µl of Buffer (Kapa Biosystems), 1.25 µl of 10 mM dNTPs (Invitrogen), 1.25 µl of each primer (Integrated DNA Technologies), 0.125 µl Taq polymerase (Kapa Biosystems), 2.5 µl of Bovine Serum Albumin (BSA)(20 mg/ml)(Thermo Scientific), 14.5 µl of dd H_2_O and 1 µl of DNA. PCR conditions were as follows: initiation at 95 °C for 5 min, followed by 35 cycles of 95 °C for 30 s, 50 °C for 1 min and 72 °C for 1 min, with a final extension for 10 min at 72 °C. Samples which showed a clear band on a safeview stained agarose gel after electrophoresis were then subject to enzymatic clean-up prior to sequencing using a mixture of 0.05 µl ExoI, 0.1 µl TSAP and 4.85 µl H_2_O, added to 20 µl of PCR product and then incubated at 37 °C for 15 min followed by 80 °C for 15 min.

Samples were subsequently sent for commercial Sanger sequencing (Macrogen). Forward and reverse strands were aligned using Geneious version 10.2.3^29^, and the consensus sequences were initially analysed using BLAST (https://blast.ncbi.nlm.nih.gov/Blast.cgi). Following this the corresponding aligned sequence from the top 100 BLAST hits were downloaded in fasta format and added to an alignment containing the four sequences for the Galana widehead catfish. The alignment file was then uploaded to the IQ-TREE web server for model selection (http://iqtree.cibiv.univie.ac.at/),^30^ and the web server of the RAxML program (https://raxml-ng.vital-it.ch)^31^ was used to generate an ML tree, with 100 bootstrap replicates performed.

## Results

### MinION sequence analysis

**Figure 2:**
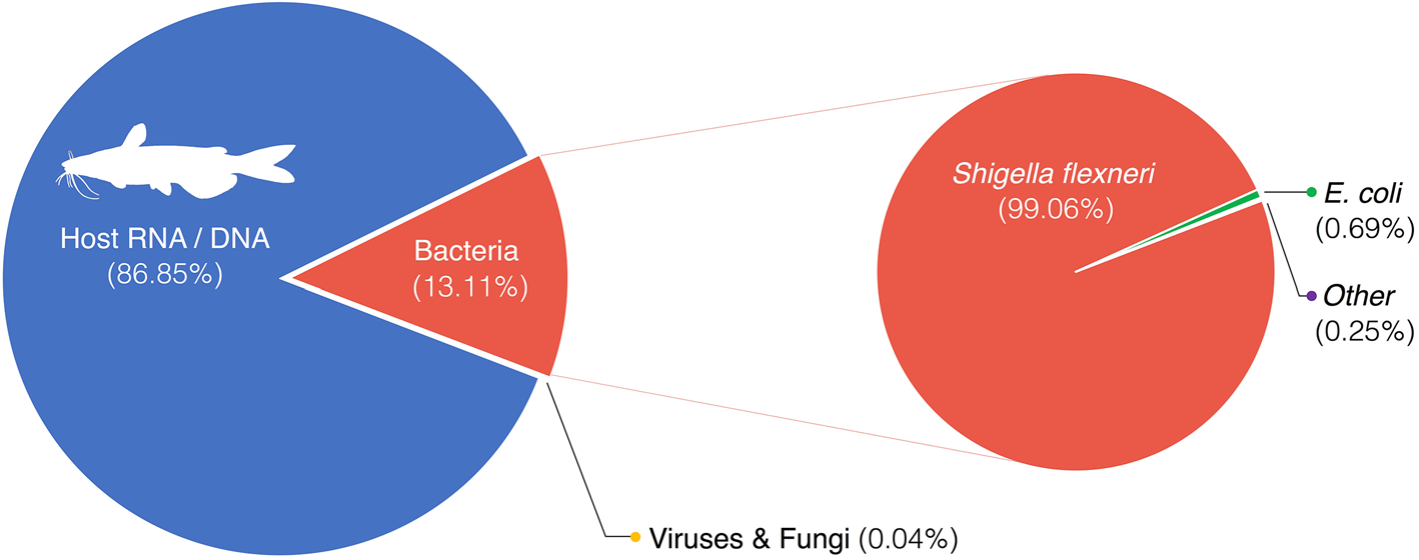
Taxon of the 59,316 reads which were successfully mapped. Host RNA is based on reads that mapped to the genome of yellow catfish. The species breakdown of the reads which mapped to bacterial sequences is also given.

Following adaptor removal, 240,429 reads remained, of which 68,908 were successfully mapped to one of the reference sequences using NanoPipe^27^. Of the 68,908 mapped reads, 59,849 mapped to the proxy host (Fig. 2), 9031 mapped to bacterial sequences, 22 mapped to fungal sequences, and 6 mapped to viral sequences. The majority of the 9031 bacterial reads (99.06%, 8,938 reads) (Fig. 2) mapped to the genome of *Shigella flexneri*, specifically to a predicted response regulator (Genbank Accession: CP045522.1, position 4,265,154—4,265,537) which shares protein homology with the two component response regulator gene *DpiA* in *Shigella boydii*^32^. Further investigation of the viral and fungal mapped reads revealed these to be ambiguous, and no bacteriophages were detected.

### Host *COI* analysis

Clean, unambiguous Sanger sequences were generated for all four widehead catfish samples, with all samples sharing the same haplotype. A BLAST search of the consensus sequence revealed the closest match to be a sequence for *Bathybagrus tetranema* (Accession number: HG803463)^33^ at 93.75% similarity. The consensus sequence matched 91.89% to an existing *Clarotes laticeps* on Genbank (Accession number: HG803491)^33^.

**Figure 3:**
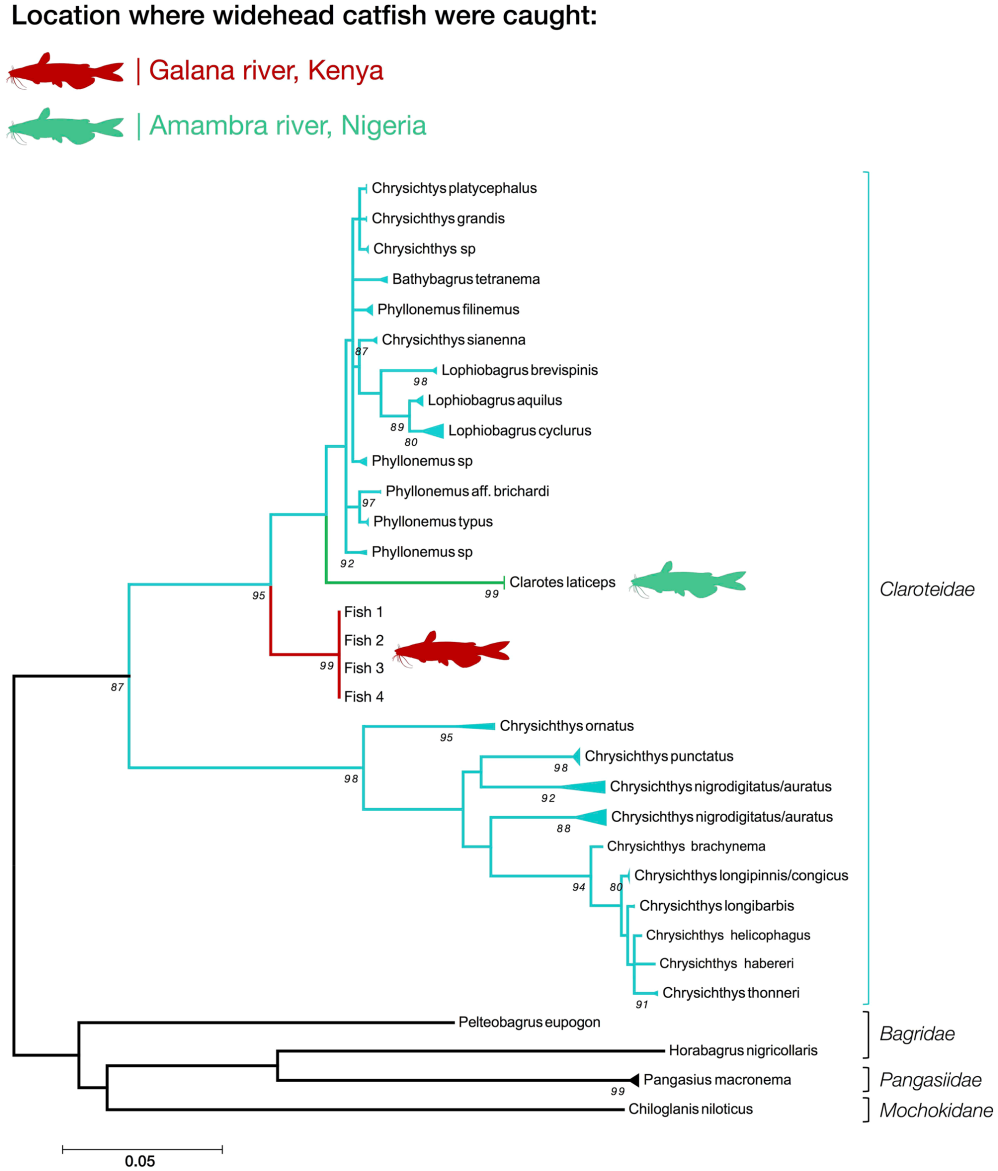
The ML tree generated using the catfish *COI* sequences. Highlighted in red are the *COI* sequences of the widehead catfish collected from the Galana river. Highlighted in green are *COI* sequences corresponding to widehead catfish collected in the Amambra river, Nigeria.

The maximum likelihood tree (Fig. 3) showed that whilst the host catfish sequence is not closely related to any existing catfish sequences, it does cluster within the family *Claroteidae*. The Galana widehead catfish show a deep split from the widehead catfish sequenced from Nigeria (Fig. 3), and show a basal split near the base of the *Claroteidae* cluster.

## Discussion

Metagenomic approaches using second generation sequencing platforms have been used previously to identify foodborne pathogens directly from a source animal^34^, however it would appear that this study represents the first time a metagenomic approach using nanopore sequencing has been used to detect a foodborne bacterial pathogen directly from an animal. Previous studies have shown the effectiveness of MinION’s nanopore sequencer as a tool for sequencing foodborne bacterial pathogens^35–37^, but these studies have relied on bacteria that were isolated from a food source and then cultured prior to sequencing. It is estimated that approximately 99% of prokaryotes are unable to be cultured in the laboratory with currently available methods^38^, which highlights the importance of developing diagnostic workflows which are culture independent, such as the approach outlined in this study.

The identification of the presence of *Shigella flexneri* in the catfish in this study highlights the potential of nanopore metagenomics as a relatively simple and effective way to detect human pathogens in one test. The causative agent of human shigellosis, *Shigella* is a genus of gram-negative bacteria that cause diarrhoea and dysentery, being a major cause of moderate-to-severe diarrhoea in sub-Saharan Africa^39^. Annually, *Shigella* is estimated to cause 600,000 deaths from 80 to 165 million cases worldwide^40^. In the developing world, *S. flexneri* is the predominant cause of shigellosis, with *S. sonnei* being the predominant strain in industrialised countries^40^. The faecal-oral route is the primary way by which *Shigella* spreads, with transmission also documented via contaminated food, drinking water and flies^41^. The *S. flexneri* gene that the majority of the reads mapped to, *DpiA,* has been shown to interfere with plasmid maintenance when overexpressed, inducing the SOS response^42^, which is a bacterial response that promotes dormancy in unfavourable environmental conditions^43^. The overabundance of the DpiA gene could imply that the bacteria were in a dormant state, but as other genes known to be involved in the SOS response were not detected with this approach, this cannot be confirmed. It may be the case that DpiA is expressed at much higher levels than the other genes in the SOS response, and a deeper sequencing effort might reveal further genes being expressed. Additionally the overexpression of DpiA has been shown to be part of the bacterial defence following exposure to β-lactam antibiotics in *E. coli*^44^, which could imply the presence of antimicrobial resistance as seen with *Shigella* spp. in other water sources^18^.

It is unclear how the catfish became carriers of *S. flexneri*, so it can only be speculated how the bacteria may have been introduced to the fish based on the known biology of the pathogen. Although the *Shigella* RNA was extracted from the heart tissue, we believe in fact it was present in the intestinal tract of the catfish, as whilst the fish were dissected in a sterilised tray, clean gloves were worn and utensils were flamed before each dissection, in the process of initially opening the fish the intestines were significantly nicked, meaning the heart samples were likely contaminated with DNA from material within the intestines. The primary hosts of *S. flexneri* are humans and primates^45^, but it has also been detected in rabbits^46^, cattle^47^, pigs^48^ and chicken^45^. Previous studies have also shown that bacterial pathogens can be introduced to fish in water bodies via the faeces of cattle, birds and wild animals^3,10–13,16^. This could imply that *S. flexneri* was introduced to the river either via the large herds of cattle which are brought to the river to drink, or through the various wild animals that use the river, with species such as hippopotamus and elephant known to be sources of other bacterial diseases such as anthrax, brucellosis, tetanus and salmonellosis (hippopotamus)^49^ and tuberculosis (elephant)^50^, and subsequently ingested in sediment by the bottom feeding catfish. In addition to the potential animal sources listed, a number of villages located approximately 150 km upstream of the sampling site, on the western side of Tsavo East National Park, are suspected to be primary sources of untreated sewage into the Galana river (John Byrne, University College Dublin, pers. comm.).

The sequence analysis of the host catfish *COI* also revealed that it was not the species *Clarotes laticeps* as suggested based on morphology, but the phylogeny does suggest the fish belongs in the family *Claroteidae*. The species *C. laticeps* is found naturally in West Africa, the Nilo-Sudanese basin and East Africa^8,26,51,52^, and the other *C. laticeps* sequence included in the analysis (Fig. 3) was collected in the Amambra river in Nigeria^53^ which is ~ 4000 km away from the Galana river, and the two rivers are separated by at least two major watersheds. According to both Okeyo^54^ and Seegers et al.^26^, and based on morphology, the only member of the family *Claroteidae* found in the Galana river is *C. laticeps*, however Seegers et al.^26^ added the caveat “*the taxonomic status of the Kenyan populations is uncertain and needs detailed study*”. This would suggest that the fish examined in this study were in fact the fish that to date have been classified as *C. laticeps* based on morphology, but now the genetic data have revealed that this population of catfish are likely a cryptic species. Based on these findings we tentatively suggest a new species name *Clarotes kambare*, or Kenyan widehead catfish, with “kambare” being the Swahili word for catfish, although further in-depth morphological and genetic work will be needed to clarify the unique taxonomic status of this population of fish. Further studies are also required to determine the full geographic location of this species and its conservation status.

In conclusion, this study has highlighted how a shotgun metatranscriptomic approach can be used to identify human pathogens in wild caught fish, and how it may highlight the transmission potential of enteropathogens in non-host animals. Further research is needed going forward to explore the potential benefits of this approach over the presence/absence results of conventional PCR assays or *16S* sequencing, as while we did highlight the presence of one gene involved in the bacterial SOS response, there are multiple genes involved which were not detected. Furthermore, the potential identification of the host as a cryptic species highlights the need for further populations of wild harvested fish to be characterised genetically, as unknowingly managing a species complex as one species may lead to severe threats to local cryptic species, in addition to masking potential disease risks in the cryptic species.

## Data Availability

The raw fastq sequences generated during the MinION sequencing are available on NCBI GenBank under the accession number PRJNA785244, and the fasta sequences for the catfish *COI* sequences are available under the GenBank accession numbers OM176588—OM176591.

## References

[1] Andersen KG, Rambaut A, Lipkin WI, Holmes EC, Garry RF (2020). The proximal origin of SARS-CoV-2. Nat. Med..

[2] Warren CJ, Sawyer SL (2019). How host genetics dictates successful viral zoonosis. PLoS Biol..

[3] McCoy E (2011). Foodborne agents associated with the consumption of aquaculture catfish. J. Food Prot..

[4] Mukwabi DM, Otieno SO, Okemo PO, Odour RO, Agwanda B (2019). Parasites infesting Nile tilapia grown in aquaculture systems in Kenya. Livest. Res. Rural Dev..

[5] Novoslavskij A (2016). Major foodborne pathogens in fish and fish products: A review. Ann. Microbiol..

[6] Allen T (2017). Global hotspots and correlates of emerging zoonotic diseases. Nat. Commun..

[7] Riede, K. Global register of migratory species: From global to regional scales: Final report of the R&D-Projekt 808 05 081. *Federal Agency for Nature Conservation*. (2004)

[8] Risch LM, Paugy D, Lévêque C, Teugels GG (2003). Claroteidae. The Fresh and Brackish Water Fishes of West Africa.

[9] Risch, L. M. Bagridae. In *Check-List of the Freshwater Fishes of Africa (CLOFFA)* (ed. Daget, J., Gosse, J. P., & Thys van den Audenaerde, D. F. E.) Vol. 2, 2–35. (ISNB, Brussels; MRAC, Tervuren; and ORSTOM, Paris, 1986)

[10] Berg RW, Anderson AW (1972). Salmonellae and Edwardsiella tarda in gull feces: A source of contamination in fish processing plants. Appl. Microbiol..

[11] Mikaelian I, Daignault D, Duval MC, Martineau D (1997). Salmonella infection in wild birds from Quebec. Can. Vet. J..

[12] Koonse B, Burkhardt W, Chirtel S, Hoskin GP (2005). Salmonella and the sanitary quality of aquacultured shrimp. J. Food Prot..

[13] Jones K (2001). Campylobacters in water, sewage and the environment. J. Appl. Microbiol..

[14] Wyatt LE, Nickelson R, Vanderzant C (1979). Occurrence and control of Salmonella in freshwater catfish. J. Food Sci..

[15] Lowry PW, McFarland LM, Threefoot HK (1986). Vibro hollisae septicemia after consumption of catfish. J. Infect. Dis..

[16] Tuyet DTN (2006). Enteropathogenic Escherichia coli o157 in Bangui and N’Goila, Central African Republic: A brief report. Am. J. Trop. Med. Hyg..

[17] Kotloff KL (2019). The incidence, aetiology, and adverse clinical consequences of less severe diarrhoeal episodes among infants and children residing in low-income and middle-income countries: a 12-month case-control study as a follow-on to the Global Enteric Multicenter Study (GEMS). Lancet Glob. Health.

[18] Shahin K, Bouzari M, Wang R, Khorasgani MR (2019). Distribution of antimicrobial resistance genes and integrons among Shigella spp. isolated from water sources. J. Glob. Antimicrob. Resist..

[19] Kinge CW, Mbewe M (2010). Characterisation of Shigella species isolated from river catchments in the North West province of South Africa. S. Afr. J. Sci..

[20] He, F. *et al*. Shigellosis outbreak associated with contaminated well water in a rural elementary school: Sichuan Province, China, June 7–16, 2009. *PLoS ONE*, **7**, e47239 (2012). 10.1371/journal.pone.0047239 PMC3468462 23071767

[21] Austin B (2019). Methods for the diagnosis of bacterial fish diseases. Mar. Life Sci. Technol..

[22] Noone JC, Helmersen K, Leegaard TM, Skråmm I, Aamot HV (2021). Rapid diagnostics of orthopaedic-implant-associated infections using nanopore shotgun metagenomic sequencing on tissue biopsies. Microorganisms.

[23] Yahara K (2021). Long-read metagenomics using PromethION uncovers oral bacteriophages and their interaction with host bacteria. Nat. Commun..

[24] Leggett RM (2020). Rapid MinION profiling of preterm microbiota and antimicrobial-resistant pathogens. Nat. Microbiol..

[25] Reddington K (2020). Metagenomic analysis of planktonic riverine microbial consortia using nanopore sequencing reveals insight into river microbe taxonomy and function. GigaScience.

[26] Seegers L, De Vos L, Okeyo DO (2003). Annotated checklist of the freshwater fishes of Kenya (excluding the lacustrine haplochromines from Lake Victoria). J. East Afr. Nat. Hist..

[27] Shabardina V (2019). NanoPipe—a web server for nanopore MinION sequencing data analysis. GigaScience.

[28] Ivanova NV, Zemlak TS, Hanner RH, Hebert PD (2007). Universal primer cocktails for fish DNA barcoding. Mol. Ecol. Notes.

[29] Kearse M (2012). Geneious Basic: An integrated and extendable desktop software platform for the organization and analysis of sequence data. Bioinformatics.

[30] Kalyaanamoorthy S, Minh BQ, Wong TK, von Haeseler A, Jermiin LS (2017). ModelFinder: Fast model selection for accurate phylogenetic estimates. Nat. Methods.

[31] Stamatakis A, Hoover P, Rougemont J (2008). A rapid bootstrap algorithm for the RAxML web servers. Syst. Biol..

[32] Pao GM, Saier MH (1995). Response regulators of bacterial signal transduction systems: Selective domain shuffling during evolution. J. Mol. Evol..

[33] Peart CR, Bills R, Wilkinson M, Day JJ (2014). Nocturnal claroteine catfishes reveal dual colonisation but a single radiation in Lake Tanganyika. Mol. Phylogenet. Evol..

[34] Grützke J (2019). Fishing in the soup–pathogen detection in food safety using metabarcoding and metagenomic sequencing. Front. Microbiol..

[35] Taylor TL (2019). Rapid, multiplexed, whole genome and plasmid sequencing of foodborne pathogens using long-read nanopore technology. Sci. Rep..

[36] Sadek M (2020). First genomic characterization of blaVIM-1 and mcr-9-coharbouring Enterobacter hormaechei isolated from food of animal origin. Pathogens.

[37] Yang M (2020). Direct metatranscriptome RNA-seq and multiplex RT-PCR amplicon sequencing on Nanopore MinION–promising strategies for multiplex identification of viable pathogens in food. Front. Microbiol..

[38] Schloss PD, Handelsman J (2005). Metagenomics for studying unculturable microorganisms: Cutting the Gordian knot. Genome Biol..

[39] Mani S, Wierzba T, Walker RI (2016). Status of vaccine research and development for Shigella. Vaccine.

[40] Bowen A (2016). Chapter 3: Infectious diseases related to travel. The Yellow Book: Health Information for International Travel.

[41] Ashkenazi S (2004). Shigella infections in children: New insights. Seminars in Pediatric Infectious Diseases.

[42] Miller C, Ingmer H, Thomsen LE, Skarstad K, Cohen SN (2003). DpiA binding to the replication origin of Escherichia coli plasmids and chromosomes destabilizes plasmid inheritance and induces the bacterial SOS response. J. Bacteriol..

[43] Žgur-Bertok D (2013). DNA damage repair and bacterial pathogens. PLoS Pathog..

[44] Memar MY (2020). The central role of the SOS DNA repair system in antibiotics resistance: A new target for a new infectious treatment strategy. Life Sci..

[45] Shi R (2014). Pathogenicity of Shigella in chickens. PLoS ONE.

[46] Jianjun J (2005). Isolation and identification of rabbits Shigella dysenteriae in a large scale warren. J. Anhui Agric. Sci..

[47] Zhu Z, Cao M, Zhou X, Li B, Zhang J (2017). Epidemic characterization and molecular genotyping of Shigella flexneri isolated from calves with diarrhea in Northwest China. Antimicrob. Resist. Infect. Control.

[48] Maurelli AT (1998). Shigellainfection as observed in the experimentally inoculated domestic pig, Sus scrofa domestica. Microbial. Pathog..

[49] Dudley JP (2016). Carnivory in the common hippopotamus Hippopotamus amphibius: Implications for the ecology and epidemiology of anthrax in African landscapes. Mamm. Rev..

[50] Murphree R, Warkentin JV, Dunn JR, Schaffner W, Jones TF (2011). Elephant-to-human transmission of tuberculosis, 2009. Emerg. Infect. Dis..

[51] Kapetsky, J. M., & Petr, T. Status of African reservoir fisheries. *FAO* (1984).

[52] Eccles DH (1992). FAO species identification sheets for fishery purposes. Field Guide to the Freshwater Fishes of Tanzania.

[53] Nwani CD (2011). DNA barcoding discriminates freshwater fishes from southeastern Nigeria and provides river system-level phylogeographic resolution within some species. Mitochondrial DNA.

[54] Okeyo DO (1998). Updating names, distribution and ecology of riverine fish of Kenya in the Athi-Galana-Sabaki River drainage system, Naga. ICLARM Q..

